# Posterior reversible encephalopathy syndrome after CD19 chimeric antigen receptor therapy for B-acute lymphoblastic leukemia: case report

**DOI:** 10.3389/fonc.2025.1639892

**Published:** 2025-09-08

**Authors:** Rohan Dipesh Agarwal, Ranju Kunwor, Kavya Sudanagunta

**Affiliations:** ^1^ Saint Louis University, St. Louis, MO, United States; ^2^ Department of Hematology Oncology, Saint Louis University, St. Louis, MO, United States; ^3^ Department of Radiology, Saint Louis University, St. Louis, MO, United States

**Keywords:** chimeric antigen receptor therapy (CAR-T), immune effector cell-associated neurotoxicity syndrome, cerebrovascular dysfunction, acute lymphoblastic leukemia, posterior reversible encephalopathy syndrome

## Abstract

Posterior reversible encephalopathy syndrome (PRES) is a neurologic condition characterized by distinctive radiologic findings and altered mental status, often associated with chemotherapy or systemic endothelial dysfunction. To date, there have been no documented cases of PRES occurring after chimeric antigen receptor T cell therapy (CAR-T) for B-cell acute lymphoblastic leukemia (B-ALL). We describe the case of a 30-year-old female patient with relapsed B-ALL who received brexucabtagene autoleucel and subsequently developed a progressively worsening cytokine release syndrome and immune effector cell-associated neurotoxicity syndrome. On day 15 post-infusion, she developed acute encephalopathy and hypertension. The brain MRI revealed new, symmetric bilateral cortical and subcortical T2/fluid-attenuated inversion recovery hyperintensities consistent with a diagnosis of PRES. Treatment with multiple courses of anti-seizure medications, anti-cytokine therapy, and high-dose pulse corticosteroids led to complete clinical recovery and resolution of the imaging abnormalities. To our knowledge, this represents the first reported case of PRES following CD19-directed CAR-T for B-ALL. This case highlights the potential for PRES as a complication of CAR-T mediated by cytokine-induced cerebrovascular endothelial injury. It also raises the possibility that a prior history of vasculitis, such as lymphomatoid granulomatosis, may predispose patients to an elevated risk of developing CAR-T-associated PRES.

## Introduction

Chimeric antigen receptor T cell therapy (CAR-T) represents one of the first commercially successful applications of cellular immunotherapy for B-cell malignancies. It has demonstrated high rates of remission and overall survival, particularly in cases of refractory disease treated with CD19-directed approaches (1). While these therapies have revolutionized the management of aggressive hematologic malignancies, a comprehensive understanding of their potential adverse effects remains critical.

Cytokine release syndrome (CRS) is the most frequently encountered side effect of CAR-T treatment, affecting up to 89% of patients (15). CRS is characterized by a systemic inflammatory response manifesting as fever, hypotension, and, in extreme cases, hypoxemia. Immune effector cell-associated neurotoxicity syndrome (ICANS), a significant complication, presents with varying degrees of encephalopathy, aphasia, and focal neurological deficits and may progress to seizures or cerebral edema (2). Both ICANS and CRS are closely monitored with standardized grading tools—vital signs for CRS and immune effector cell-associated encephalopathy (ICE) score for ICANS—and treated with interleukin inhibitors (3).

In rare instances, patients may develop posterior reversible encephalopathy syndrome (PRES), a distinct clinical–radiologic diagnosis characterized by acute encephalopathy, hypertension, and vasogenic edema affecting the parieto-occipital regions on MRI (4). PRES associated with CAR-T is infrequently reported and may not respond to conventional management strategies for ICANS or CRS (4). We report the case of a 30-year-old female patient with relapsed B-cell acute lymphoblastic leukemia (B-ALL) who developed grade 3 ICANS and hemodynamic instability following treatment with a de-intensified dose of brexucabtagene autoleucel, an anti-CD19 CAR-T product. The patient demonstrated full clinical and radiologic recovery following an extended course of high-dose corticosteroids. This case highlights a rare but important manifestation of neurotoxicity in the context of CAR-T and underscores the need for heightened clinical vigilance for atypical presentations.

## Case presentation

The patient is a 30-year-old woman with trisomy 21, hypothyroidism, and relapsed B-ALL. She was initially diagnosed at 6 years and treated with POG protocol #9905 (14). However, the course was complicated with recurrent HSV stomatitis and Epstein–Barr virus (EBV)-related grade 3 pulmonary lymphomatoid granulomatosis of the left lung and maxillary sinus. These complications were treated with one course of cyclophosphamide/vincristine, followed by six courses of rituximab, cyclophosphamide, doxorubicin, vincristine, etoposide, and prednisolone (R-CHOEP), and she subsequently achieved complete remission.

At age 30, she was hospitalized with acute onset of fatigue, anorexia, fever, diffuse petechiae, and pancytopenia. A bone marrow biopsy confirmed the relapse of Philadelphia-like B-ALL with complex molecular cytogenetics: CRLF2 rearrangement, CRLF2 exon 5 deletion, JAK2 gain, 9q34 abnormality, and mutations in ETV6, KRAS, and NRAS. Due to high chemosensitivity, she underwent re-induction on a single de-intensified dose of 6-mercaptopurine, vincristine, methotrexate, and prednisolone (POMP), along with pegaspargase. At 2 months after induction, the bone marrow biopsy and flow cytometry revealed minimal residual disease of 0.59% in total nucleated cells, with negative cerebrospinal fluid, which prompted the decision to initiate CAR-T.

The baseline MRI obtained prior to CAR-T cell therapy was unremarkable. Following cell collection for CAR-T, the patient underwent lymphodepleting chemotherapy with cyclophosphamide and fludarabine. During this period, she was treated for influenza virus with oseltamivir and initiated on institutional standard prophylaxis for gram-negative bacteria, *Pneumocystis jirovecii* pneumonia (PJP), herpes simplex virus (HSV), fungal infections, and seizure prevention.

On day 0, she received an infusion of 100 × 10^6^ CAR-T cells. That evening, she developed a fever of 102°F, consistent with grade 2 CRS with ICE score of 10/10. Her empiric antibiotic coverage was broadened to cefepime for neutropenia prophylaxis, and her CRS resolved with acetaminophen and negative infectious workup.

On day 5, she experienced acute abdominal pain, anorexia, fever, and hemodynamic instability with poor response to fluid resuscitation. This clinical decline, consistent with grade 3 CRS and ICE scores of 10/10, prompted her transfer to the intensive care unit for vasopressor support. She remained at neurologic baseline and was treated with tocilizumab (8 mg/kg) and dexamethasone (10 mg) per institutional protocol. By day 7, her hypotension resolved, and CRS was downgraded to grade 1. A trial of dexamethasone and acetaminophen de-escalation was attempted at that time.

On the morning of day 8, the patient’s condition declined again, with a fever of 103.3°F, tachypnea, hypotension, and progressive somnolence. This was consistent with grade 3 CRS and grade 3 ICANS, with an ICE score of 0/10. In response, intravenous methylprednisolone (500 mg) and anakinra were initiated. The brain MRI and fundoscopic exam were unremarkable; however, continuous electroencephalogram (cEEG) revealed seizure-like activity and evidence of encephalopathy. Seizure prophylaxis (levetiracetam) was increased, and antibiotic coverage was escalated to meropenem.

Over the course of days 10 and 11, the patient made neurological and hemodynamic recovery to her baseline mental status, allowing the discontinuation of anakinra. On day 12, an attempt was made to taper methylprednisolone to dexamethasone; however, this resulted in acute bradycardia with recurrence of somnolence. Consequently, methylprednisolone was re-administered at 1 g per day over 2 days. On day 14, a second taper to dexamethasone was reattempted.

**Figure 1:**
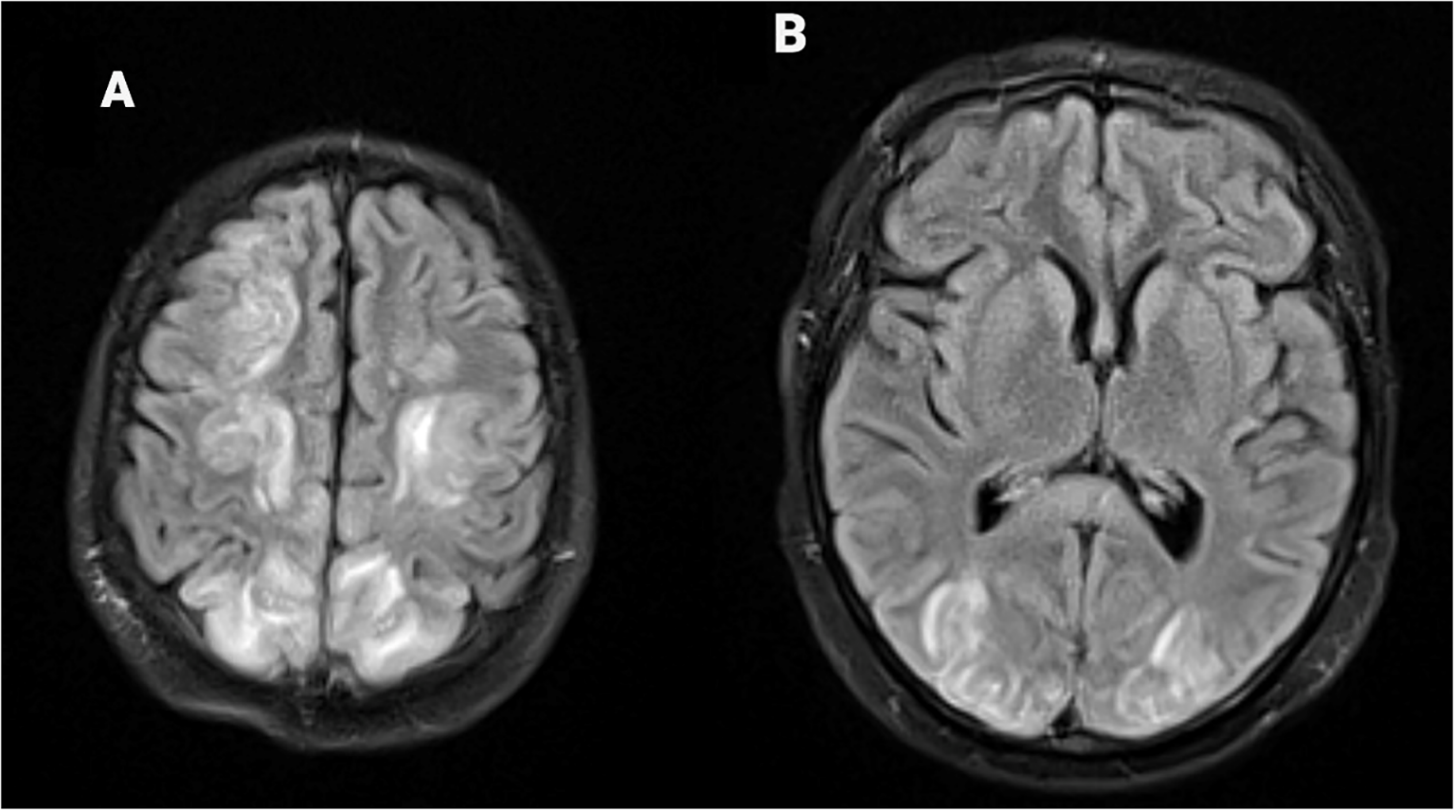
Axial MR brain **(A, B)** FLAIR demonstrates bilateral symmetrical widespread cortical/subcortical T2/FLAIR with high signal intensity, suggesting vasogenic edema in the frontal, parietal region **(A)** and occipital lobes **(B)**.

**Figure 2:**
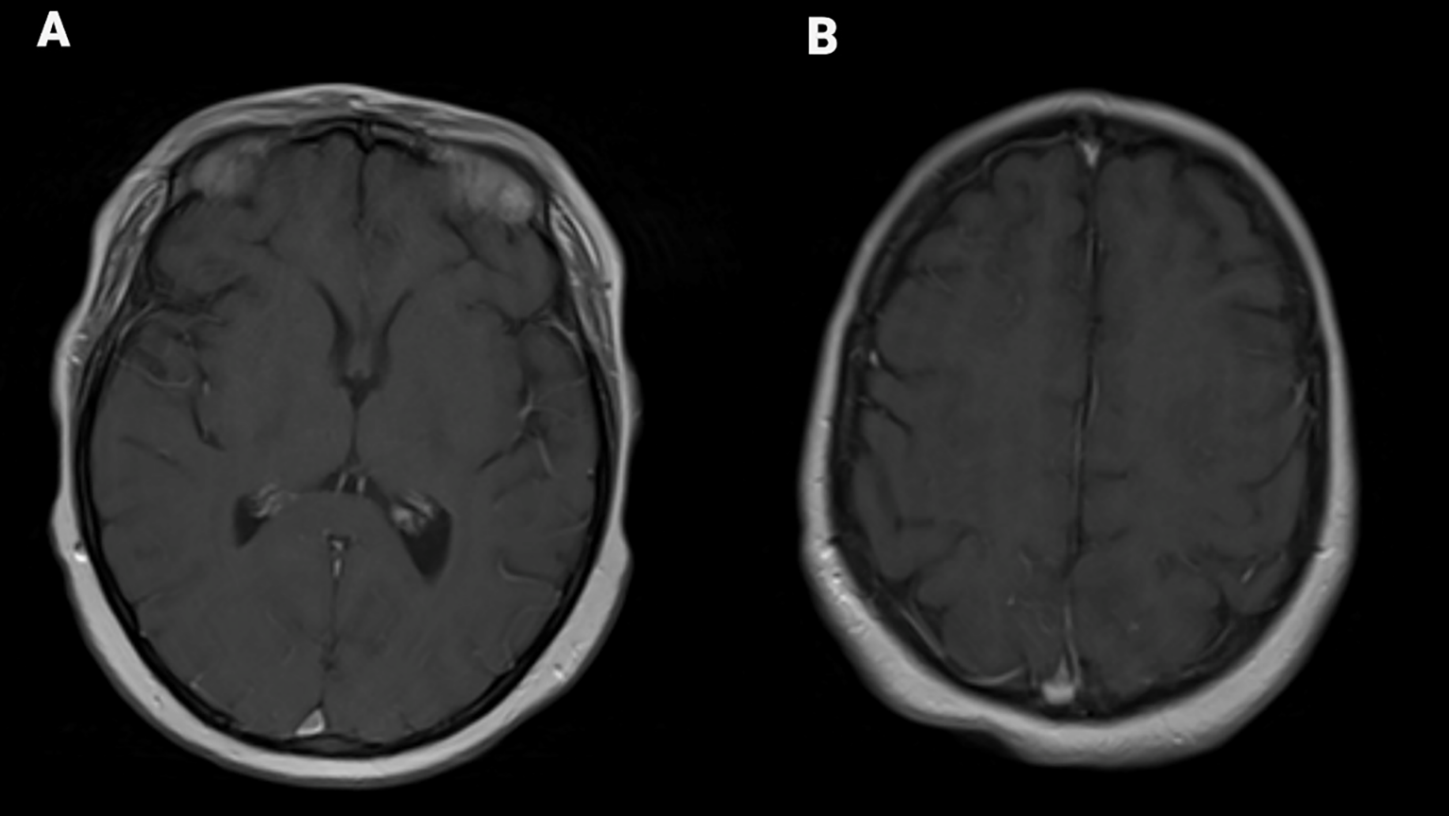
Axial MR brain T1 post-contrast images through the frontal, parietal region **(A)** and occipital lobes **(B)** show no post-contrast enhancement.

By day 15, the patient exhibited new-onset tearfulness, profound fatigue, and generalized weakness, including difficulty in holding her phone or a sandwich. She was hemodynamically stable (grade 0 CRS), but her ICE score was 9/10, consistent with grade 1 ICANS. A repeat brain MRI with and without contrast revealed new, symmetric T2/fluid-attenuated inversion recovery (FLAIR) hyperintensities in the bilateral cortical and subcortical parieto-occipital and frontal lobes (**Figures 1A, B**), without associated enhancement (**Figures 2A, B**). These findings were consistent with vasogenic edema localized to watershed regions, diagnostic of posterior reversible encephalopathy syndrome (PRES). Following this result, a 5-day pulse course of methylprednisolone (1 g per day) and anakinra was initiated.

On day 16, the patient developed agitation, hypertension of 187/109 mmHg, and lactic acidosis of 7.4 mmol/L, attributed to high-dose corticosteroid therapy and the ongoing effects of PRES. The symptoms were managed supportively. On day 19, steroids were successfully transitioned to dexamethasone (8 mg three times daily). By day 21, the patient had returned to her neurological and physical baseline, with exception of muscular deconditioning. She was discharged on day 22 on a 10-day taper course of prednisone and seizure prophylaxis with levetiracetam.

**Figure 3:**
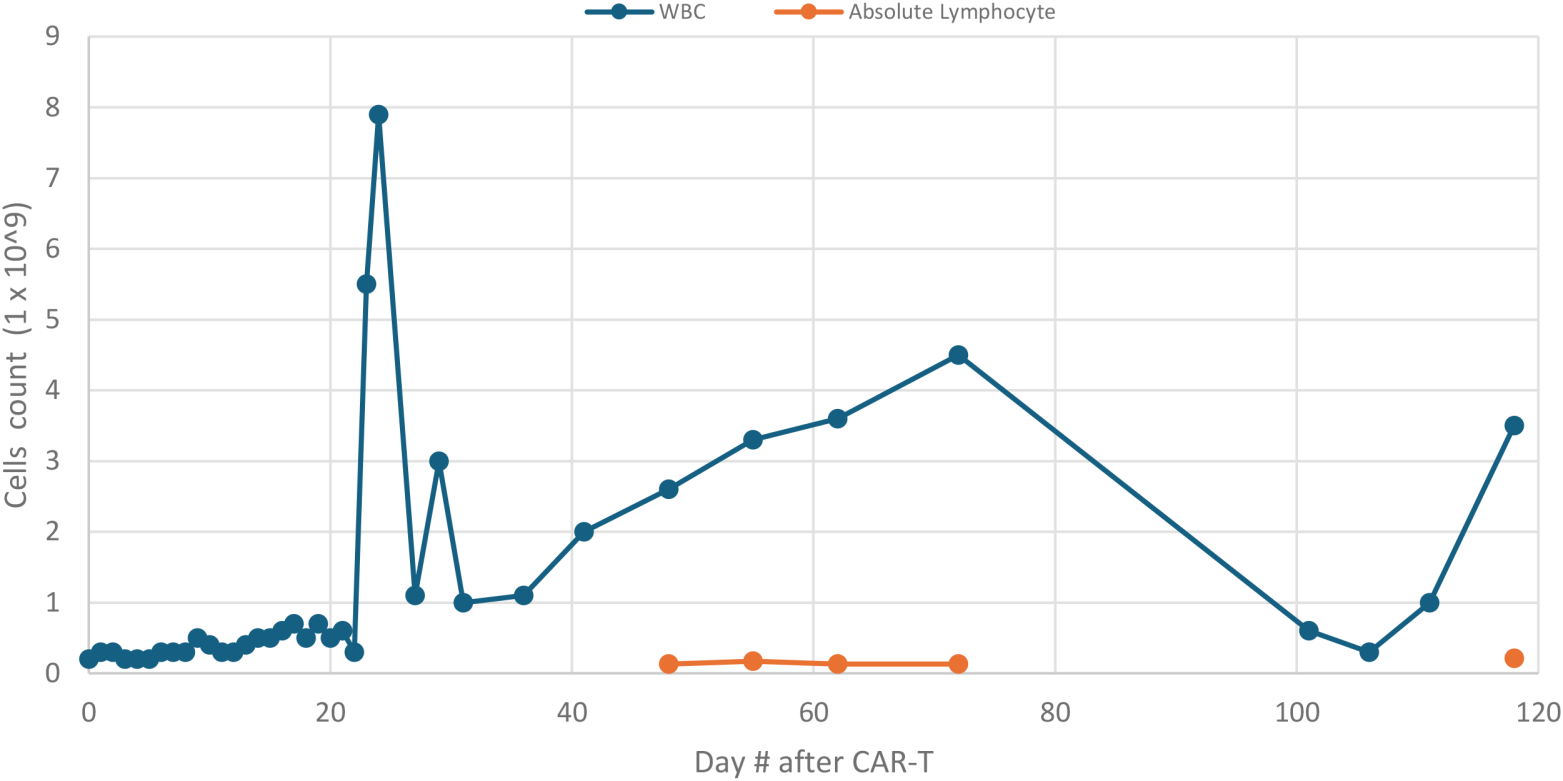
Total leukocyte (WBC) and absolute lymphocyte count by day post-CAR-T. The figure demonstrates leukopenia during inpatient hospitalization for CAR-T (days 0–22) with an increase after G-CSF and IVIG administration in outpatient monitoring phase (days 23–70), followed by repeat leukopenia during viral infection (days 71–110) and eventual recovery.

**Figure 4:**
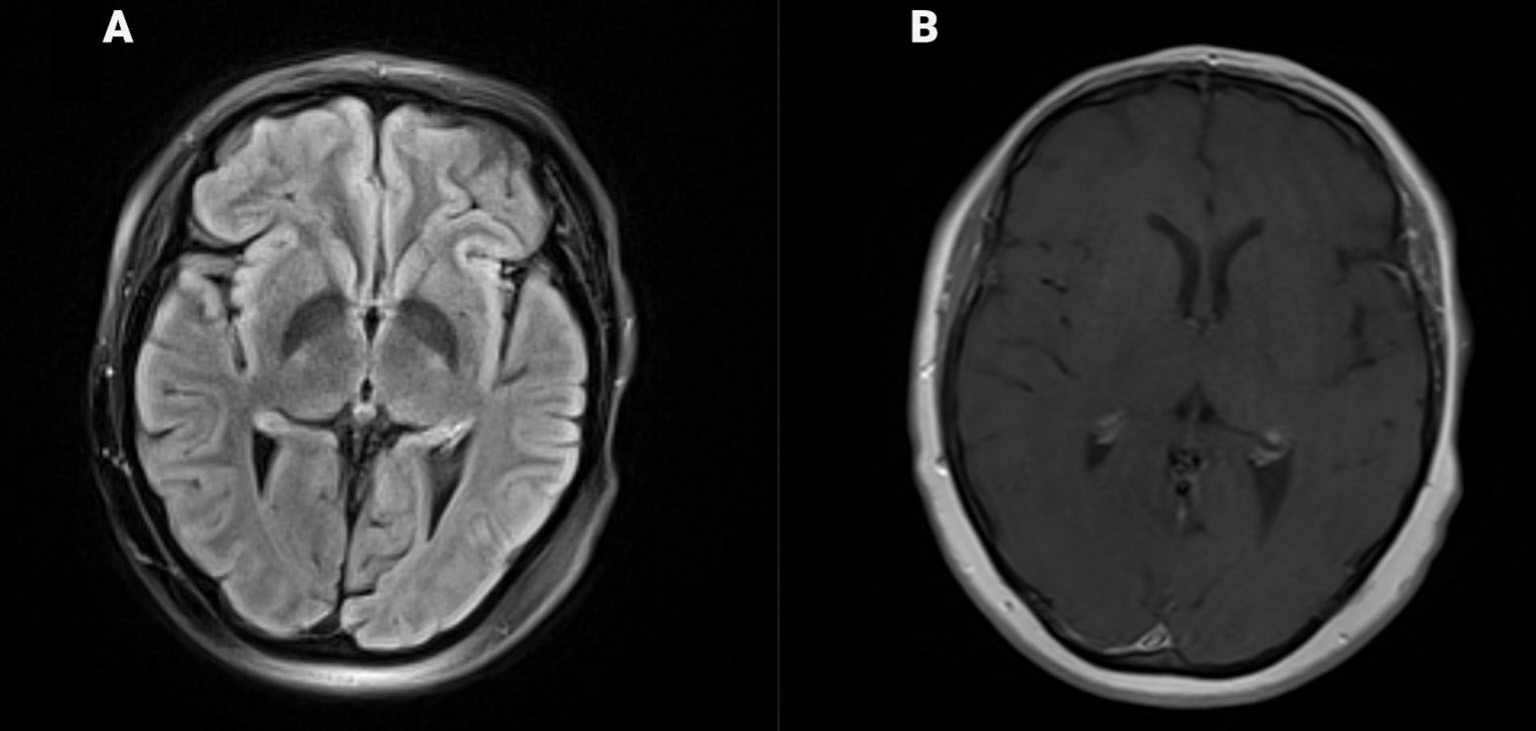
Axial MR image FLAIR demonstrates no T2/FLAIR signal intensity, suggesting the resolution of vasogenic edema in a previously seen region **(A)**, with continued absence of enhancement within regions on axial MR brain T1 post-contrast images **(B)**.

The patient was also given granulocyte colony-stimulating factor (G-CSF) to boost leukocyte counts at discharge day, with positive cell count response (**Figure 3**). She continued institutional antimicrobial and seizure prophylaxis with intravenous immunoglobulin (IVIG) infused monthly. A bone marrow biopsy with flow cytometry performed on day 40 showed no evidence of malignancy and confirmed minimal residual disease negativity. During this period, she reported improved appetite, ambulation, and physical activity. A follow-up brain MRI on day 69 demonstrated the complete resolution of the previously noted vasogenic edema (**Figures 4A, B**). The patient had persistent leukopenia by day 70, followed by acute-onset cough, myalgia, rhinorrhea, and diarrhea. This was confirmed to be parainfluenza on viral panel, with CT imaging revealing no new gastrointestinal processes. The patient’s symptoms and leukopenia both improved by day 120 (**Figure 3**).

## Discussion

**Figure 5:**
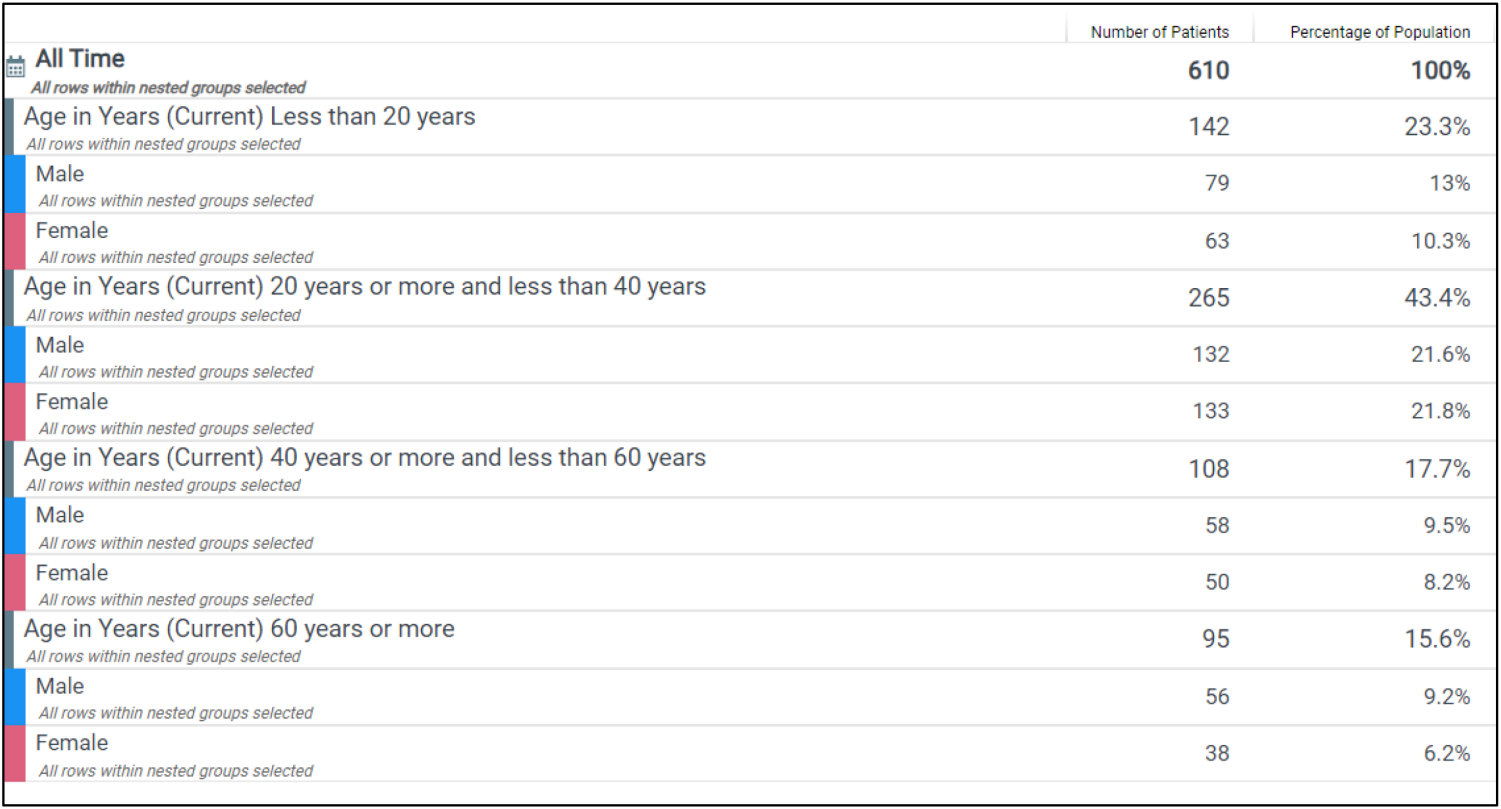
Relapsed and primary B-ALL population by age range of diagnosis (years) and legal sex within the hospital system.

Our patient was a 30-year-old woman with relapsed acute B-cell lymphoblastic leukemia. An analysis of our hospital system’s demographic data revealed 610 patients with either primary or relapsed B-ALL. Of these, the largest group—43%—comprised patients between 20 and 40 years old, with an equal distribution of male and female patients (**Figure 5**). This contrasts from the SEER B-ALL national registry, which reports the most common age group at diagnosis as 1–4 years, highlighting a distinct local patient population (16).

**Figure 6:**
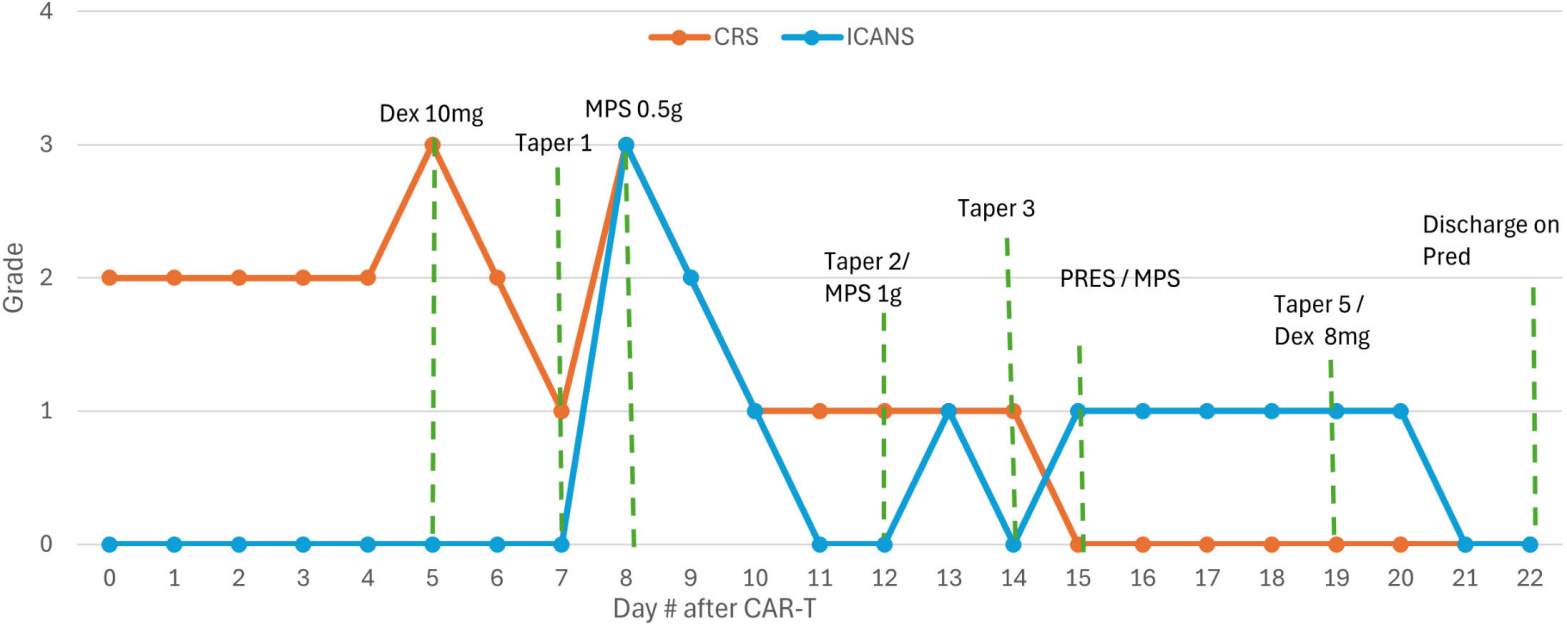
Patient’s hospital course displayed by CRS/ICANS grade day post-CAR therapy. Taper represents each day on which an attempt was made to de-escalate a preceding steroid treatment, typically leading to worsening grades of ICANS or CRS until resolution with Taper 5. Dex represents IV dexamethasone. MPS represents IV methylprednisolone. PRES represents posterior reversible encephalopathy, diagnosed on day 15. Pred represents final oral prednisone taper that the patient was discharged on.

This case was notable for recurrent episodes of proinflammatory decompensation following CD19 CAR-T therapy, leading to PRES. The patient initially developed grade 1 CRS, requiring treatment escalation from acetaminophen to dexamethasone, with multiple courses of methylprednisolone in the setting of ICANS. Each episode was precipitated by attempts to taper corticosteroids (**Figure 6**). A 5-day course of high-dose-pulse methylprednisolone and anakinra brought symptom resolution, suggesting that cytokine-mediated neurotoxicity culminating in radiologically confirmed PRES was the primary driver of her clinical deterioration.

ICANS is believed to result from cytokine-induced endothelial disruption of the blood–brain barrier (BBB), leading to both cytotoxic edema and white matter demyelination (5–7). Interestingly, in this case, no abnormalities were observed on brain MRI during the episode of grade 3 ICANS. This aligns with prior reports that ICANS can present with a range of imaging findings—from no abnormalities to periventricular white matter hyperintensities, microhemorrhages, leptomeningeal enhancement, or severe cerebral edema (8). Given the severity of ICANS observed, brain parenchymal changes would typically be expected.

The subsequent appearance of symmetric bilateral T2/FLAIR hyperintensities in the frontal, parietal, and occipital lobes—along with clinical deterioration—aligns more consistently with PRES than with a delayed ICANS manifestation, although radiologic overlap has been reported (9, 10). The timing of PRES onset (day 15 post-infusion) mirrors another reported case of PRES following BCMA-targeted CAR-T therapy in a patient with multiple myeloma (4). This comparison supports that PRES may represent a downstream complication of ICANS mediated by cytokine-induced endothelial dysfunction, inflammation, and impaired cerebral autoregulation, particularly in regions supplied by “watershed” posterior circulation. Notably, in our patient, MRI findings of PRES preceded the onset of hypertensive urgency, further supporting a localized cerebral endothelial mechanism.

Additional predisposing factors may have contributed to PRES development (10). The patient required two courses of norepinephrine for CRS-related hypotension, potentially inducing transient cerebral hypoperfusion, followed by compensatory vasoconstriction and edema. Concurrent high-dose corticosteroids may have also elevated the mean arterial blood pressure and contributed to BBB disruption. She had a remote history of high-grade lymphomatoid granulomatosis—a necrotizing EBV-associated vasculitis involving the pulmonary blood vessels (11). Though this disease was in remission at the time of CAR-T, a residual vasculopathy may have heightened her vulnerability to CAR-T-induced endothelial injury. Infectious causes were deemed unlikely due to persistent blood cultures and broad-spectrum antimicrobial coverage. PRES from lymphodepleting chemotherapy is less probable. Though chemotherapeutics like cyclophosphamide and fludarabine are associated with PRES, the temporal delay in symptoms and absence of baseline imaging findings make this less likely as well (12).

To date, PRES following CAR-T remains poorly characterized, with only a handful of documented cases. One such report involved a 55-year-old patient with refractory multiple myeloma who developed PRES on BCMA CAR-T at a higher dose (200 × 10^6^ cells) and responded to cyclophosphamide refractory to corticosteroids. In contrast, our patient responded to high-dose methylprednisolone alone. A cohort study of 100 patients with relapsed/refractory diffuse large B-cell lymphoma (DLBCL) treated with CD19 CAR-T axi-cel found that 68 developed ICANS. Of these, less than one quarter had abnormal brain MRI findings, and only two were diagnosed with PRES (13). The imaging patterns in these cases resembled those seen in our patient. Notably, worsening MRI findings in the cohort study correlated with poor neurological outcomes and were associated with reduced overall and progression-free survival. This underscores the importance of prompt recognition and management.

## Conclusion

To our knowledge, this is the first reported case of posterior reversible encephalopathy syndrome (PRES) following CD19-directed CAR-T cell therapy for B-ALL successfully managed with medical therapy. This case highlights PRES as a potential downstream complication in the setting of CRS and ICANS, driven by cytokine-mediated cerebrovascular endothelial dysfunction. Resolution of symptoms and radiographic findings was achieved with repeated courses of high-dose corticosteroids, antiepileptics, anti-cytokine agents, and supportive care. These findings underscore the need for vigilance and prompt recognition and management of delayed neurotoxicity in CAR-T recipients, particularly those with predisposing vascular risk factors.

## Data Availability

The original contributions presented in the study are included in the article/Supplementary Material. Further inquiries can be directed to the corresponding author.
